# Predictive Analysis and Validation of Critical Missense SNPs of the *ABH2* Gene Using Structural Bioinformatics

**DOI:** 10.3390/ijms262311593

**Published:** 2025-11-29

**Authors:** Anastasiia T. Davletgildeeva, Timofey E. Tyugashev, Viktoriia V. Sagalakova, Mingxing Zhao, Nikita A. Kuznetsov

**Affiliations:** 1 Institute of Chemical Biology and Fundamental Medicine, Siberian Branch of Russian Academy of Sciences, 630090 Novosibirsk, Russia; tyugashev@niboch.nsc.ru (T.E.T.); zurachok@mail.ru (V.V.S.); 2 Department of Natural Sciences, Novosibirsk State University, 630090 Novosibirsk, Russia; hdzhaomingxing@163.com

**Keywords:** DNA repair, base methylation, human DNA dioxygenase ABH2, single-nucleotide polymorphism, demethylation efficiency, enzymatic activity

## Abstract

Human DNA dioxygenase ABH2 is a key enzyme of the AlkB family of Fe(II)/α-ketoglutarate-dependent oxygenases, which is specialized in removing alkyl groups from damaged DNA bases in the cell nucleus. At the same time, the occurrence of single-nucleotide polymorphisms (SNPs) in the human *ABH2* gene can lead to amino acid substitutions that, in turn, may disrupt the normal functioning of the ABH2 enzyme. Currently, databases contain information about more than 2500 nucleotide substitutions in the *ABH2* gene. Using a comprehensive bioinformatics approach, in this review, we analyzed over 200 non-synonymous ABH2 SNPs with eleven prediction programs to identify variants capable of negatively affecting its enzymatic activity. The combination of various programs with different evaluation algorithms and scoring approaches allows us to more reliably identify potentially deleterious amino acid substitutions. Moreover, the differences between the programs used allowed for comparison of their tendency to predict amino acid substitutions as deleterious. Structural analysis of the ABH2-substrate complex showed that selected functionally significant SNPs often affect the organization of the active site, reduce the efficiency of substrate binding, and/or disrupt the coordination of Fe^2+^ and α-ketoglutarate cofactors, leading to changes in catalytic efficiency. The data obtained from the conducted analysis suggest that naturally occurring polymorphisms in the *ABH2* gene found in the human population may reduce the repair efficiency of DNA dioxygenase ABH2 and, consequently, modulate susceptibility to oncogenesis and influence the effectiveness of antitumor therapy for carriers of these SNPs.

## 1. Introduction

DNA alkylation occurring under the influence of various endogenous and exogenous factors can provoke cytotoxicity and the accumulation of mutations associated with oncogenesis [1,2,3,4]. To eliminate such lesions, cells utilize various repair mechanisms, including removal of damaged bases by DNA glycosylases, direct removal of methyl groups by O^6^-methylguanine-DNA methyltransferase, or their oxidation by AlkB-like dioxygenases [2,3,5,6,7].

Dioxygenases of the AlkB family belong to Fe(II)/α-ketoglutarate(αKG)-dependent oxygenases and are widely represented in both prokaryotes and eukaryotes [8,9,10]. These enzymes catalyze the removal of alkyl groups from diverse substrates, including DNA, RNA, and proteins [5,11,12]. In mammals, nine AlkB homologs have been identified (ABH1–ABH8 and FTO), each containing a conserved dioxygenase domain [13,14,15,16]. However, their intracellular localization, substrate specificity, and biological functions differ substantially [17]. Some of the nine homologs, in particular ABH5 and FTO, have been extensively studied as RNA demethylases [18,19,20]. At the same time, for a number of enzymes, such as ABH4, ABH6, and ABH7, the protein structures and functional roles of amino acid residues forming their active sites have not been fully studied yet [17].

## 2. Functional Properties of ABH2

**Figure 1:**
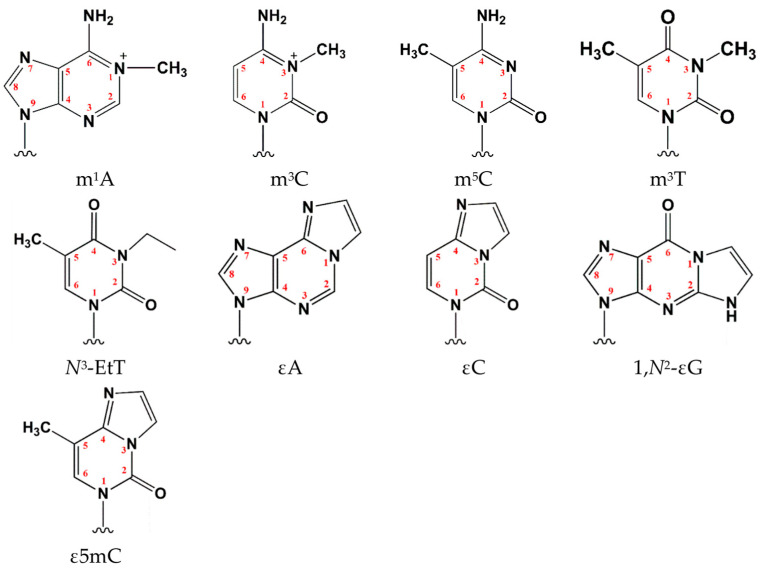
Alkylated DNA bases that are substrates for human DNA dioxygenase ABH2. Alkylated modifications of adenosine: *N*^1^-methyladenosine (m^1^A) and 1,*N*^6^-ethenoadenosine (εA); alkylated modifications of cytidine: *N*^3^-methylcytidine (m^3^C), 5-methylcytidine (m^5^C), 3,*N*^4^-etheno-5-methylcytosine (ε5mC), and 3,*N*^4^-ethenocytidine (εC); alkylated modifications of thymidine: *N*^3^-methylthymidine (m^3^T), and *N*^3^-ethylthymidine (*N*^3^-EtT); alkylated modification of guanosine—1,*N*^2^-ethenoguanosine (1,*N*^2^-εG).

ABH2 is of particular interest as one of the key enzymes responsible for repairing alkylated DNA in the nucleus. The enzymes ABH2 and ABH3 reside within the same clade of the phylogenetic tree. For ABH2, unlike other human AlkB homologs, a fairly broad spectrum of oxidizable substrates is known, including at least nine distinct alkylated DNA bases: *N*^1^-methyladenosine (m^1^A), *N*^3^-methylcytidine (m^3^C), *N*^3^-methylthymidine (m^3^T), *N*^3^-ethylthymidine (*N*^3^-EtT), 1,*N*^6^-ethenoadenosine (εA), 3,*N*^4^-ethenocytidine (εC), 1,*N*^2^-ethenoguanosine (1,*N*^2^-εG), 5-methylcytidine (m^5^C), and 3,*N*^4^-etheno-5-methylcytosine (ε5mC) (Figure 1) [21,22,23,24,25,26]. ABH2 clearly prefers to oxidize lesions within double-stranded DNA (dsDNA), in contrast to its close homolog ABH3 [5,27,28,29,30].

Analysis of the known crystal structures of *E. coli* AlkB and its human homologs revealed that these enzymes share a common dioxygenase domain with a double-stranded β-helix fold (“jelly-roll” or DSBH), composed of eight β-strands that surround the active site [28,29,31]. In contrast to the prokaryotic AlkB, which primarily forms contacts with the damaged DNA strand and flips the damaged base into the active site, ABH2 employs an additional specific hydrophobic “finger,” consisting of residues V101, F102, and G103. This finger intercalates into the bound DNA duplex, filling the cavity created by the base flipped-out into the active site [28,29]. The preference of ABH2 for double-stranded substrates is due to additional contacts with the DNA strand complementary to the damaged one. These contacts are provided by a positively charged RKK loop (R241, K242, and K243) and a long flexible loop containing DNA-binding residues R198, R203, and K205 [28,29,30]. Coordination of the Fe^2+^ ion required for catalysis in the ABH2 active site, as in other family members, is achieved by the H171, D173, and H236 triad [32].

## 3. Impact of Single-Nucleotide Polymorphisms (SNPs) on ABH2 Activity

Numerous scientific data accumulated over the past two decades demonstrate that disruptions in the regulation of DNA methylation and repair of alkylated lesions (both due to changes in expression levels and as a result of the appearance of mutant forms of enzymatic components) may be associated with the development of various diseases, including the onset and progression of oncological diseases and various neurological disorders [11,17]. Human AlkB homologs are of particular interest in this area due to their key role in two interconnected processes: DNA damage repair and carcinogenesis. Numerous studies demonstrate that these proteins are often overexpressed in various types of malignant neoplasms and can reduce the effect of alkylating drugs used in chemotherapy, such as temozolomide [33,34]. It is known, for example, that AlkB homologs ABH8 and ABH3 are already being used as targets in antitumor therapy [35,36]. The dioxygenase FTO, the ninth human AlkB homolog discovered, is associated with obesity development [37,38,39,40], type 2 diabetes [41,42], Alzheimer’s disease [43,44], and nonalcoholic steatohepatitis [45]. There is evidence that ABH1 plays a certain role in the development of gastric cancer and glioblastoma [46,47]. Examples exist of the discovery of mutant forms and/or altered expression of both ABH2 and ABH3 in several different human oncological diseases [11,48]. At the same time, compared to other components of the methylation/demethylation system, there is not as much data on the role of ABH2 in oncogenesis and the development of other diseases.

As one of the key participants in the repair system for alkylated DNA lesions, ABH2, which efficiently removes such common modifications as m^1^A or m^3^C in dsDNA, thus plays an important role in protecting genetic material from the damaging effects of endogenous and exogenous alkylating agents [13,49,50]. Increasing evidence indicates that altered expression of DNA dioxygenase ABH2 in patients with various malignant neoplasms affects disease development. For instance, it was shown that the sensitivity of non-small cell lung cancer cell lines to chemotherapy increases with ABH2 downregulation [51]. Conversely, the opposite effect was demonstrated for ABH2 upregulation in human glioblastoma cell lines [33]. At the same time, altered ABH2 expression registered in pediatric brain tumor samples suggests a certain role for ABH2 in counteracting some types of cancer [52,53,54]. ABH2 downregulation inhibits epithelial–mesenchymal transition [53], and a similar regulatory effect was demonstrated in colorectal cancer (CRC) cell lines, where ABH2 knockdown inhibited proliferation and invasive capacity of CRC cells [55].

SNPs are the most common form of genetic variation in humans [56]. Genetic variations caused by SNPs, particularly nonsynonymous SNPs in coding regions, occurring in protein-coding regions, alter the natural amino acid residues of enzymes, potentially causing structural and functional changes. Some amino acid substitutions caused by SNPs may not significantly affect protein function, while others may be detrimental to efficient enzymatic function performance due to structural changes and/or effects on functional properties.

Despite growing interest in the role of ABH2 in carcinogenesis, current scientific data on the association of known polymorphic variants of this enzyme with disease development and chemoresistance formation remain extremely limited. Analysis of the global literature reveals only isolated studies devoted to this problem. In particular, Cetica et al. identified an I141V missense mutation in ABH2 in a glioma patient, with I141 localized in a highly conserved region of the protein preserved in *E. coli*, mice, and humans [52]. Fu et al. also investigated two polymorphic ABH2 variants containing amino acid substitutions A9V or Q10K. Both substitutions are localized in the domain responsible for ABH2 binding to proliferating cell nuclear antigen (PCNA), which may be important for regulating ABH2 repair activity in tumor cells. Moreover, both are associated with oncological diseases according to the Catalogue of Somatic Mutations in Cancer (COSMIC) database. Indeed, the authors showed that both these substitutions, while having minimal effect on catalytic activity targeting methylated dsDNA substrates, reduce (in the case of A9V substitution) or, conversely, significantly increase ABH2 affinity for PCNA in the case of the Q10K polymorphic variant [57]. A recent study analyzing the relationship between hereditary mutations in DNA repair system genes and predisposition to early-onset lung adenocarcinoma also identified an association of the frameshift mutation p.E35fs in the *ABH2* gene with increased risk of this pathology [58].

The totality of currently available data on the potential role of human DNA dioxygenase ABH2 in oncological disease development and chemoresistance, as well as the paucity of information about the effect of existing polymorphic variants on the catalytic efficiency of this enzyme, makes it an attractive object for research in this area.

## 4. Prediction of SNP Consequences on Protein Function

Modern researchers now have numerous bioinformatics tools that allow prediction of the possible effects of SNPs on protein functions to varying degrees. In this work, we analyzed known polymorphisms in the human Fe(II)/αKG-dependent dioxygenase ABH2 to identify substitutions that may negatively affect the functionality of this enzyme. The primary set of ABH2 SNPs was obtained from the NCBI dbSNP database (https://www.ncbi.nlm.nih.gov/variation/view/, accessed on 29 July 2025). According to the NCBI database, more than 2500 nucleotide substitutions in the *ABH2* gene are currently registered, more than half of which are localized in the intronic region. In the coding regions of the gene, 231 variations (nucleotide substitutions) were found that lead to changes in the class of the corresponding amino acid residue and, consequently, may lead to changes in ABH2 enzyme functions. All SNPs that lead to amino acid residue substitutions (nonsynonymous or missense mutations) were analyzed using eleven programs employing different algorithms to assess the presumed ability of substitutions to lead to protein function disruption: SIFT (Sorting Intolerant From Tolerant) [59,60], PolyPhen2 (Polymorphism Phenotyping) [61], CADD (Combined Annotation-Dependent Depletion) [62], REVEL (Rare Exome Variant Ensemble Learner) [63], MetaLR [64], AlphaMissense [65], PROVEAN (Protein Variation Effect Analyzer) [66], WS-SNPs&GO [67], PANTHER (Protein Analysis Through Evolutionary Relationships) [68], PredictSNP [69], and PhD-SNP (Predictor of human Deleterious Single-Nucleotide Polymorphisms) [70]. A detailed description of algorithms for predicting SNP effects on enzyme functioning using these software resources is presented in the Supplementary Materials.

Using multiple methods to predict SNP consequences on ABH2 enzyme functions is appropriate because different methods have certain advantages and disadvantages. The combination of available predictive resources can significantly increase the reliability of obtained predictions, especially in situations where the negative effect of SNPs is close to the threshold value and may be false. At the same time, using this approach does not eliminate possible prediction errors, although it minimizes them compared to using the single program as well as a smaller set of programs. Therefore, the predictive data obtained using this approach allows us to identify a range of potentially important amino acid substitutions caused by SNPs for further research using bioinformatics and experimental approaches. Methods such as REVEL, MetaLR, and PredictSNP use an integrative approach, combining predictions from other computational methods, which increases their accuracy [69,71,72,73,74,75]. However, the spectrum of SNP variants for which consequences can be predicted using these integrative programs is relatively limited. SIFT, PANTHER, and PROVEAN mainly focus on the conservation of substituted amino acid residues among orthologs [64,66,68,76,77,78,79,80]. Their effectiveness is relatively high, but the reliability of results depends in part on the number of available sequences for each individual protein. CADD and PolyPhen2 consider both data on the conservation of analyzed amino acid residues and structural data, allowing assessment of the functional role of individual residues at a deeper level [62,81]. Programs such as AlphaMissense, WS-SNPs&GO, and PhD-SNP, through the application of deep machine learning, allow expansion of the range of parameters considered in prediction, adding data such as information about the immediate environment of the substituted amino acid residue and its molecular functions [65,67,82].

**Figure 2:**
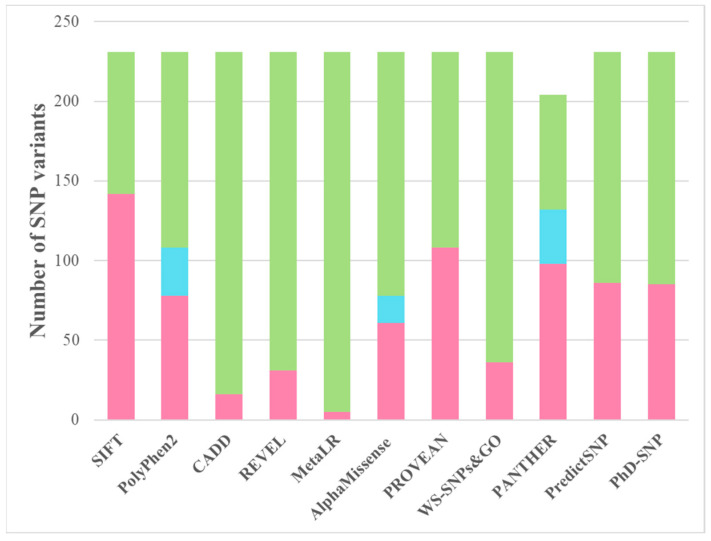
Histogram of the distribution of SIFT, PolyPhen2, CADD, REVEL, MetaLR, AlphaMissense, PROVEAN, WS-SNPs&GO, PANTHER, PredictSNP, and PhD-SNP assessments for deleterious (pink), possibly deleterious * (blue), and neutral (green) variants. * This classification level is used only by PolyPhen2, AlphaMissense, and PANTHER.

When analyzing all non-synonymous SNP variants of ABH2 registered in the NCBI dbSNP database, different distributions of assessments between deleterious and neutral variants were obtained for the 11 programs used (Figure 2). The range of the proportion of deleterious variants from the total pool of non-synonymous SNPs from 2% (5 out of 231 for MetaLR) to 62% (142 out of 231 for SIFT) and 65% (132 out of 204 for PANTHER) is related to the above-described features of prediction construction and, accordingly, different degrees of accuracy and probability of classifying a particular variant as deleterious.

**Figure 3:**
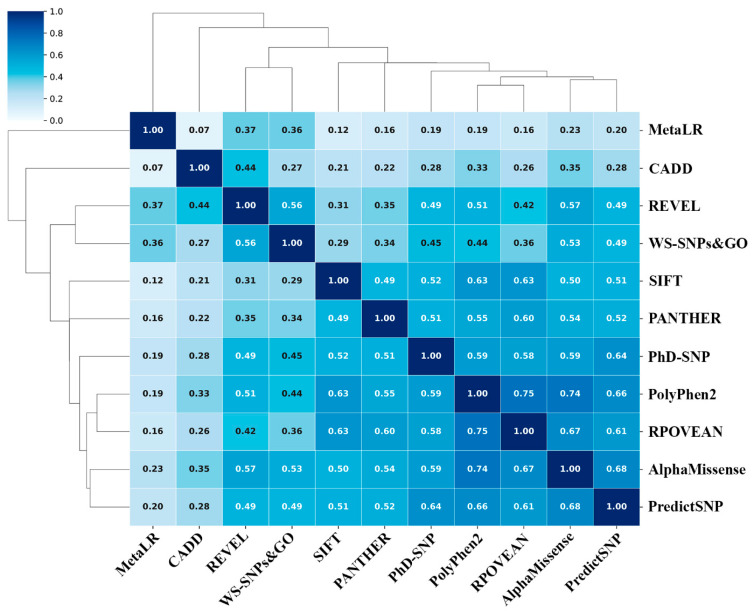
Correlation coefficients between 11 methods based on analysis of 204 non-synonymous ABH2 SNP variants.

To assess the correlation of prediction results between any two programs used, we calculated the Spearman rank correlation coefficient (ρ) based on prediction scores from all 11 methods for 204 non-synonymous ABH2 SNP variants (limited due to PANTHER). As shown in Figure 3, the highest correlation was found between PolyPhen2 and PROVEAN (ρ: 0.75), and PolyPhen2 and AlphaMissense (ρ: 0.74). AlphaMissense prediction scores with PROVEAN and PredictSNP, as well as PolyPhen2 and PredictSNP, also had high positive correlation compared to other methods. In the latter case, the correlation level is partly related to the fact that PredictSNP, being an integrative method, also considers PolyPhen2 data. MetaLR prediction scores have the lowest correlation with other methods, which is explained by the smallest number of SNP variants classified by this program as deleterious.

Thus, analysis of all ABH2 enzyme SNP variants using a set of modern available bioinformatics resources allowed prediction of negative effects for SNP-associated amino acid substitutions. The combination of different resources should significantly increase the reliability of predictions.

## 5. Selection of the Most Significant SNP-Associated Amino Acid Substitutions

After testing in the above-mentioned programs, we selected those polymorphic forms of ABH2 in which amino acid residue substitution presumably had a significant negative effect on protein functions, according to predictions from at least eight of the eleven programs used (SIFT: deleterious/tolerated, PolyPhen2: probably and possibly damaging/benign, CADD: likely deleterious/likely benign, REVEL: likely disease-causing/likely benign, MetaLR: damaging/tolerated, AlphaMissense: likely pathogenic/ambiguous/likely benign, PROVEAN: damaging/neutral, WS-SNPs&GO: disease/neutral, PANTHER: probably damaging/possibly damaging/likely benign, PredictSNP: deleterious/neutral, and PhD-SNP: deleterious/neutral). Table 1 presents the polymorphic ABH2 variants selected as a result of the conducted analysis.

Thirty-three such ABH2 SNPs were identified (Table 1). These cover 25 distinct residue positions because four residues (W134, S186, R248, and R254) each have two alternative substitutions, and two positions (G190 and R193) have three alternative substitutions. In addition to functional predictions, databases collecting disease associations were queried to identify links between ABH2 polymorphisms and cancer. cBioPortal (https://www.cbioportal.org/, accessed on 29 July 2025) integrates genomic and clinicopathologic data from large cancer genomics projects. BioMuta (https://hive.biochemistry.gwu.edu/biomuta/, accessed on 29 July 2025) is a database of single-nucleotide variants and disease associations mapped to RefSeq [83,84,85]. Among the 33 selected SNPs, at least four variants may be associated with cancer: G190S with endometrioid carcinoma of the uterus (cBioPortal, BioMuta), R193T with invasive ductal carcinoma of the breast (cBioPortal), M226T with liver cancer (BioMuta), and R254C with chromophobe renal cell carcinoma (cBioPortal, BioMuta).

## 6. Functional Roles of the Identified Residues

**Figure 4:**
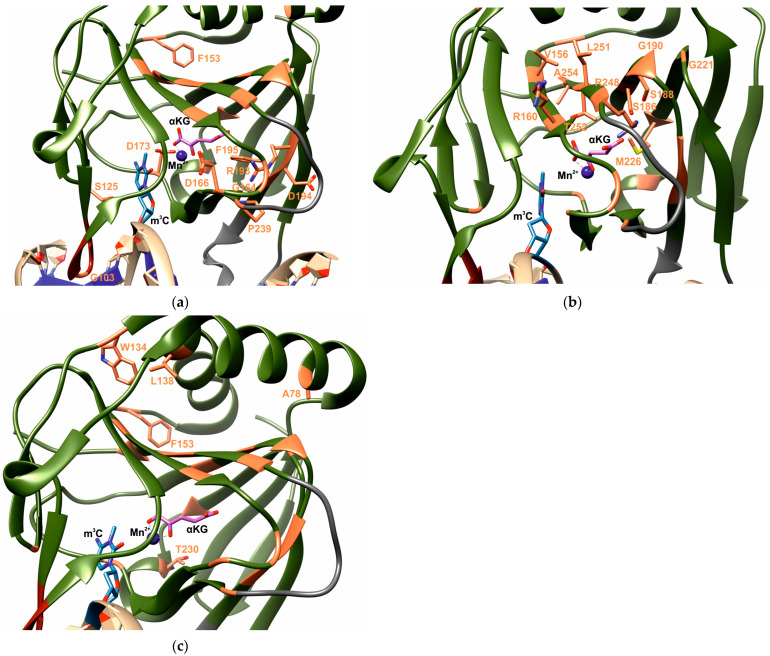
Positions of residues whose substitutions are predicted to have high negative impact on the ABH2 structure (PDB ID 3RZJ). Selected residues are shown in orange across three views (**a**–**c**), to simplify their visual localization. Mn^2+^ is shown in blue, αKG in pink. Note: Alanine is present at position 254 in this structure, but UniProt (https://www.uniprot.org/uniprotkb/Q6NS38/entry, accessed on 26 August 2025) indicates arginine at position 254 in ABH2.

Analysis of the DNA dioxygenase ABH2 structure in complex with substrate (Figure 4) allowed us to hypothesize about the role of SNP-induced amino acid residue substitutions selected in this work in maintaining both overall protein structure and in DNA substrate recognition and catalytic reaction processes.

The A78T substitution may disrupt positioning of amino acid residue H220, including through destruction of hydrogen bonds between its side chain and the side chains of residues D79 and G190. However, the overall amino acid environment in the A78 region allows accommodation of the more voluminous threonine side chain without introducing significant perturbations to the overall protein fold.

Substitution of residue G103, which participates in forming loop L1, through which the enzyme checks base pair stability in DNA substrates [50], with arginine may lead to disruption of complementary DNA strand binding and thus potentially reduce ABH2 catalytic activity.

S125, together with amino acid residues Y122 and F124, forms functional loop L2, representing the “nucleotide recognition lid (NRL)” involved in substrate recognition [50]. Therefore, the S125L substitution localized in the DNA backbone binding region presumably should lead to serious disruptions in ABH2 activity due to the appearance of a voluminous hydrophobic leucine residue in this functionally important region. This assumption is additionally supported by literature data indicating that the mutant ABH2 form containing the S125A substitution is characterized by at least reduced catalytic activity, and according to other data, it completely loses activity toward both m^1^A- and m^3^C-containing dsDNA substrates [86,87].

Substitutions of amino acid residue W134 with cysteine or arginine presumably should reduce overall protein globule stability in this region, since in this case, there is loss of a large hydrophobic side chain in a hydrophobic region. The substitution with cysteine will additionally lead to loss of a hydrogen bond with the side chain of D117, which probably participates in interaction with the flexile unresolved C-terminus of ABH2. With the W134R substitution, conversely, additional hydrogen bonds arise with residue E139. In this regard, there may be competition with residue R142, which maintains a network of hydrogen bonds with E139 and D143, and the backbone oxygen atom of F153. In turn, the F153L substitution may create steric conflict with the side chain of residue F253; however, the effect of this on overall stability should be insignificant due to the possibility of compensation through displacement of the loop containing F153.

The L138P substitution leads to a turn in the corresponding α-helix due to the appearance of a proline residue in this position, which may have a significant destabilizing effect on protein structure. Nevertheless, the fact that this amino acid residue is located at the very beginning of the α-helix, as well as the fact that in the structure of ABH2 homologous proteins AlkB from *E. coli* and human ABH1, the corresponding position is occupied by a proline residue, may indicate that the enzyme can retain activity fully or at least partially with the L138P substitution.

The F153L substitution creates steric conflict with the side chain of F253, which should be accompanied by displacement of peptide backbones of interacting residues and, apparently, destabilization of the protein globule. It should be noted that among AlkB-like enzymes, only F or Y residues are found in this position, confirming the significance of a planar aromatic substituent at position 153.

Since residue V156 is highly conserved among AlkB-like enzymes, its substitution with glycine may have quite serious consequences for ABH2 activity. In addition to the expected loss of hydrophobic contacts within the β-sheet core of the protein, the V156G substitution will lead to displacement of side chains L251 and F253. The positions of side chains T252 and L157, which participate in forming the enzyme’s active site, may also change.

Introduction of the R160G substitution leads to loss of an intramolecular salt bridge with residue E86, which potentially may affect protein globule stability.

The G164D substitution should lead to reorientation of amino acid residues D163 and K242, disrupting the interaction of the latter with the DNA sugar-phosphate backbone. Moreover, introduction of a voluminous amino acid residue in this position instead of glycine will naturally lead to changes in permissible conformations of the functionally significant L4 loop.

With the D166G substitution, there is loss of intramolecular contacts with residue L162. Additionally, one can speak of possible negative effects of losing the carboxyl group in the enzyme folding region with increased content of positively charged residues overall. It should be noted that although this position is conservative among orthologs, some representatives have amino acid residues such as asparagine and glutamate in this position.

Substitution of the catalytically important amino acid residue D173, which participates in Fe^2+^ ion coordination in the ABH2 enzyme active site [88], with asparagine should lead to loss of enzyme catalytic activity. This assumption is also confirmed by several literature sources, indicating that the D173A substitution leads to almost complete loss of ABH2 enzyme activity [27,89].

Amino acid residue S186 is part of a hydrogen bond network involving the ω-carboxyl group of αKG and also enters into direct interaction with residue N250. In this regard, the S186C substitution may lead to suboptimal αKG positioning in the enzyme active site and, consequently, to reduced ABH2 catalytic activity. The substitution of S186 with aromatic phenylalanine residue will very likely lead to significant loss of enzyme catalytic activity.

The side chain of S188 forms a hydrogen bond directly with the side chain of residue R248, which forms a salt bridge with the αKG ω-carboxyl, so introduction of phenylalanine in this position presumably will lead to complete disruption of αKG coordination and appearance of an inactive mutant ABH2 form.

Amino acid residue G190 makes an important contribution to the ABH2 β-sheet core folding, so all three substitutions, G190S, G190R, and G190D, most likely introduce significant perturbation to enzyme stability, both due to the appearance of steric hindrances when introducing more voluminous side chains and differing permissible torsion angles at the β-sheet terminus.

Residue R193 is highly conserved among AlkB-like enzymes, and introduction of substitutions R193K, R193T, or R193S leads to disruption of the hydrogen bond network involving oxygen atoms of the backbones of amino acid residues Y161, H167, F195, P239, and A246. In this regard, all three substitutions may have significant effects on ABH2 protein stability. Direct effects on positioning of amino acid residue R248 and indirect effects on residue Y161, both participating in αKG coordination in the enzyme active site, are also possible [32]. Thus, mutant forms will change the structure of loop L4, affect amino acid residues Y161 and R248 coordinating the αKG ω-carboxyl group, and may influence the orientation of the L238 side chain forming the active site wall. Changes in αKG position in the active site alter the optimal position of the oxidizable bond in the nitrogenous base substrate, i.e., may affect substrate specificity. All this collectively indicates that substitutions R193K, R193S, and R193T should lead to significant reduction in enzyme activity.

The D194Y substitution disrupts the network of contacts between mobile side chains of amino acid residues D194 and R241, R215, and K243, which may negatively affect ABH2 interaction with DNA. At the same time, among orthologs, residue D194 is semi-conservative, and residues such as glutamate, asparagine, threonine, and lysine are found in its place.

The F195S substitution leads to the appearance of a new possible hydrogen bond donor for αKG in the “hydrophobic wall” region of the ABH2 active site in a relatively accessible location for the α-carboxyl group, which suggests some reduction in activity of the mutant ABH2 F195S form due to the addition of possibilities for catalytically incompetent αKG binding.

The G221R substitution potentially leads to steric conflict with the α1-helix, changing torsion angles in this region of the structure.

Despite the fact that the M226T substitution should increase the volume of the active site pocket, the position of residue M226 suggests minimal effect on overall ABH2 stability and catalytic activity, unlike the F195S substitution.

With the T230I substitution, there is loss of hydrogen bonds with the backbone of amino acid residues D66 and N319, which may lead to insignificant reduction in ABH2 protein globule folding stability.

The P239S substitution also should not have significant effect on ABH2 activity, since the serine side chain can form both hydrogen bonds with the DNA backbone and with the side chain of DNA-binding residue R241.

Since R248, as mentioned above, enters into direct interaction with αKG in the enzyme active site, substitutions of this amino acid residue with glutamine or tryptophan should have significant effect on ABH2 catalytic activity.

The L251P substitution should lead to disruption of the β-sheet structure in which this amino acid residue is localized, which, in turn, may have a strong destabilizing effect on the overall ABH2 enzyme protein globule folding.

Substitution of residue T252 with isoleucine, as in the case of R248 substitutions, leads to loss of αKG coordination [32]. Moreover, this substitution leads to loss of a hydrogen bond with another amino acid residue participating in αKG binding in the ABH2 active site, R254, which collectively should have a strong negative effect on enzyme activity.

Similarly, substitutions R254C and R254H lead to disruption of catalytically important αKG coordination, as well as loss of contact with D173 participating in Fe^2+^ ion coordination, which collectively should lead to loss of ABH2 catalytic activity.

Thus, analysis of the enzyme-substrate complex structure suggests that most predicted negative variants are related to effects on protein globule structure and/or substrate coordination in the enzyme active site. Moreover, the critical role of some residues in the catalytic process carried out by ABH2, namely S125 and D173, was experimentally confirmed by studying mutant forms of ABH2 containing substitutions of these amino acid residues to alanine [27,86,87,89]. However, in several cases, for example, A78T, L138P, M226T, or P239S, analysis of structural data did not reveal functional effects from the corresponding substitutions. In this regard, one can assume that even such a multifactorial predictive approach using a set of bioinformatics resources may give false results. Nevertheless, one can also assume that these residues may be important for coordination/regulation of DNA dioxygenase ABH2 action in the cell, for example, in interaction with histones or with regulatory proteins participating in the repair process, including PCNA, as well as other protein factors, but do not directly affect the enzyme’s functional properties.

## 7. Conclusions

DNA dioxygenase ABH2 is an important enzyme for repairing alkylated lesions in the human genome. Currently, increasing evidence indicates that disruption of ABH2 regulation in cells or the appearance of mutant forms of this enzyme may be associated with both oncological disease development and cell resistance to antitumor therapy. Changes in the efficiency of repair of alkylated DNA lesions by ABH2 in cells could result (among other factors) from naturally occurring polymorphic variants of this enzyme possessing altered catalytic activity. It is important to note that individual SNPs are associated with increased risk of oncological disease development, which emphasizes their clinical significance.

In this regard, in this work a systematic bioinformatics analysis of ABH2 protein SNP-associated amino acid substitutions was performed and the potential impact of natural substitutions on protein functional properties was assessed. The combination of eleven programs with different evaluation algorithms and scoring approaches (i) allowed us to more reliably identify potentially deleterious amino acid substitutions and (ii) revealed differences between used programs, highlighting the ability of each program to predict amino acid substitutions as deleterious. Moreover, analysis of the DNA dioxygenase ABH2 structure in complex with substrate allowed us to put forward hypotheses about molecular causes of amino acid residue substitution effects caused by SNPs. It was established that some of the resulting amino acid substitutions can disrupt native enzyme structure stability, affect the efficiency of its interaction with DNA, and negatively impact catalytic activity through effects on Fe^2+^ cofactor or αKG co-substrate binding or active site organization. Some polymorphic ABH2 variants, while preserving enzymatic activity, apparently may have altered patterns of interaction with various participants in the repair process.

Thus, the bioinformatics analysis conducted in this work suggests that single-nucleotide polymorphisms in the *ABH2* gene may have significant effects on enzyme functional properties, and further research on functional consequences of *ABH2* gene mutations occurring in the human population represents an important direction in molecular biology and medicine. Moreover, reported approach of comparative analysis, together with the description of the algorithms used by the various prediction programs, may be useful for studies of any genes of interest. In-depth study of SNP effects on enzymatic activity and protein–protein interactions can not only expand understanding of molecular mechanisms of carcinogenesis but also contribute to the development of new diagnostic and therapeutic strategies.

## Figures and Tables

**Table 1:** ABH2 SNP-induced amino acid substitutions with a high predicted probability of altering enzymatic functional activity (negative predictions by at least eight of eleven programs: SIFT, PolyPhen2, CADD, REVEL, MetaLR, AlphaMissense, PROVEAN, WS-SNPs&GO, PANTHER, PredictSNP, and PhD-SNP).

Aa substitutions	SIFT	PolyPhen2	CAAD	REVEL	MetaLR	Alpha Missense	PROVEAN	WS-SNPs&GO	PANTHER	PredictSNP	PhD-SNP
**A78T**	Del.	Pr. dam.	L. b.	L. b.	Tol.	l. p.	Dam.	Dis.	Pr. dam.	Del.	Del.
**G103R**	Del.	Pr. dam.	L. b.	L. d. c.	Tol.	l. p.	Dam.	Dis.	Pr. dam.	Del.	Del.
**S125L**	Del.	Pr. dam.	L. d.	L. d. c.	Tol.	l. p.	Dam.	N.	Pr. ben.	Del.	Del.
**W134C**	Del.	Pr. dam.	L. d.	L. d. c.	Tol.	l. p.	Dam.	Dis.	Pr. dam.	Del.	Del.
**W134R**	Del.	Pr. dam.	L. b.	L. d. c.	Tol.	l. p.	Dam.	Dis.	Pr. dam.	Del.	Del.
**L138P**	Del.	Pr. dam.	L. d.	L. b.	Tol.	l. p.	Dam.	Dis.	Pr. dam.	Del.	Del.
**F153L**	Del.	Pr. dam.	L. b.	L. d. c.	Tol.	l. p.	Dam.	N.	Pr. dam.	Del.	Del.
**V156G**	Del.	Pr. dam.	L. b.	L. d. c.	Tol.	l. p.	Dam.	Dis.	Pr. dam.	Del.	Del.
**R160G**	Del.	Pr. dam.	L. d.	L. b.	Tol.	l. p.	Dam.	N.	Pr. dam.	Del.	Del.
**G164D**	Del.	Pr. dam.	L. b.	L. b.	Tol.	l. p.	Dam.	Dis.	Pr. dam.	Del.	Del.
**D166G**	Del.	Pr. dam.	L. b.	L. d. c.	Tol.	l. p.	Dam.	Dis.	Pr. dam.	Del.	Del.
**D173N**	Del.	Pr. dam.	L. b.	L. d. c.	Dam.	l. p.	Dam.	Dis.	Pr. dam.	Del.	Del.
**S186C**	Del.	Pr. dam.	L. d.	L. d. c.	Tol.	l. p.	Dam.	N.	Pr. dam.	Del.	Del.
**S186F**	Del.	Pr. dam.	L. d.	L. d. c.	Tol.	l. p.	Dam.	N.	Pr. dam.	Del.	Del.
**S188F**	Del.	Pr. dam.	L. d.	L. d. c.	Tol.	l. p.	Dam.	Dis.	Pr. dam.	Del.	Del.
**G190S**	Del.	Pr. dam.	L. d.	L. d. c.	Tol.	l. p.	Dam.	Dis.	Pr. dam.	Del.	Del.
**G190R**	Del.	Pr. dam.	L. d.	L. d. c.	Tol.	l. p.	Dam.	Dis.	Pr. dam.	Del.	Del.
**G190D**	Del.	Pr. dam.	L. b.	L. d. c.	Tol.	l. p.	Dam.	Dis.	Pr. dam.	Del.	Del.
**R193T**	Del.	Pr. dam.	L. d.	L. d. c.	Tol.	l. p.	Dam.	Dis.	Pr. dam.	Del.	Del.
**R193S**	Del.	Pr. dam.	L. b.	L. b.	Tol.	l. p.	Dam.	Dis.	Pr. dam.	Del.	Del.
**R193K**	Del.	Pr. dam.	L. b.	L. d. c.	Tol.	l. p.	Dam.	Dis.	Pr. dam.	Del.	Del.
**D194Y**	Del.	Pr. dam.	L. d.	L. b.	Tol.	l. p.	Dam.	Dis.	Pr. dam.	Del.	Del.
**F195S**	Del.	Pr. dam.	L. d.	L. d. c.	Tol.	l. p.	Dam.	Dis.	Pr. dam.	Del.	Del.
**G221R**	Del.	Pr. dam.	L. b.	L. d. c.	Tol.	l. p.	Dam.	Dis.	Pr. dam.	Del.	Del.
**M226T**	Del.	Pr. dam.	L. b.	L. d. c.	Tol.	l. p.	Dam.	Dis.	Pr. dam.	Del.	Del.
**T230I**	Del.	Pr. dam.	L. b.	L. d. c.	Tol.	l. p.	Dam.	N.	Pr. dam.	Del.	Del.
**P239S**	Del.	Pr. dam.	L. b.	L. d. c.	Tol.	l. p.	Dam.	N.	Pr. dam.	Del.	Del.
**R248Q**	Del.	Pr. dam.	L. b.	L. d. c.	Dam.	l. p.	Dam.	Dis.	Pr. dam.	Del.	Del.
**R248W**	Del.	Pr. dam.	L. b.	L. d. c.	Dam.	l. p.	Dam.	Dis.	Pr. dam.	Del.	Del.
**L251P**	Del.	Pr. dam.	L. b.	L. d. c.	Tol.	l. p.	Dam.	Dis.	Pr. dam.	Del.	Del.
**T252I**	Del.	Pr. dam.	L. b.	L. d. c.	Tol.	l. p.	Dam.	N.	Pr. dam.	Del.	Del.
**R254C**	Del.	Pr. dam.	L. b.	L. d. c.	Dam.	l. p.	Dam.	Dis.	Pr. dam.	Del.	Del.
**R254H**	Del.	Pr. dam.	L. b.	L. d. c.	Dam.	l. p.	Dam.	Dis.	Pr. dam.	Del.	Del.

Del.—deleterious, Pr. dam.—probably damaging, L. b.—likely benign, L. d.—likely deleterious, L. d. c.—likely disease-causing, Tol.—tolerated, Dam.—damaging, Dis.—disease, l. p.—likely pathogenic, N.—neutral.

## Data Availability

The original contributions presented in this study are included in the article/Supplementary Materials. Further inquiries can be directed to the corresponding author.

## Supplementary Materials

The following supporting information can be downloaded at: https://www.mdpi.com/article/10.3390/ijms262311593/s1.

## Abbreviations

The following abbreviations are used in this manuscript:

1,*N*^2^-εG	1,*N*^2^-ethenoguanosine
εA	1,*N*^6^-ethenoadenosine
ε5mC	3,*N*^4^-etheno-5-methylcytosine
εC	3,*N*^4^-ethenocytidine
m^5^C	5-methylcytosine
αKG	α-ketoglutarate
DSBH s	Double-stranded β-helix fold
*N*^3^-EtT	*N*^3^-ethylthymidine
m^1^A	*N*^1^-methyladenosine
m^3^C	*N*^3^-methylcytidine
m^3^T	*N*^3^-methylthymidine
SNP	Single-nucleotide polymorphism

## References

[1] Schofield C.J., Zhang Z. (1999). Structural and Mechanistic Studies on 2-Oxoglutarate-Dependent Oxygenases and Related Enzymes. Curr. Opin. Struct. Biol..

[2] Sedgwick B. (2004). Repairing DNA-Methylation Damage. Nat. Rev. Mol. Cell Biol..

[3] Mishina Y., Duguid E.M., He C. (2006). Direct Reversal of DNA Alkylation Damage. Chem. Rev..

[4] Hecht S.S. (1999). DNA Adduct Formation from Tobacco-Specific N-Nitrosamines. Mutat. Res..

[5] Fedeles B.I., Singh V., Delaney J.C., Li D., Essigmann J.M. (2015). The AlkB Family of Fe(II)/α-Ketoglutarate-Dependent Dioxygenases: Repairing Nucleic Acid Alkylation Damage and Beyond. J. Biol. Chem..

[6] Kuznetsov N.A., Kanazhevskaya L.Y., Fedorova O.S. (2021). DNA Demethylation in the Processes of Repair and Epigenetic Regulation Performed by 2-Ketoglutarate-Dependent DNA Dioxygenases. Int. J. Mol. Sci..

[7] Lindahl T., Sedgwick B., Sekiguchi M., Nakabeppu Y. (1988). Regulation and Expression of the Adaptive Response to Alkylating Agents. Annu. Rev. Biochem..

[8] Aravind L., Koonin E.V. (2001). The DNA-Repair Protein AlkB, EGL-9, and Leprecan Define New Families of 2-Oxoglutarate- and Iron-Dependent Dioxygenases. Genome Biol..

[9] Falnes P.Ø., Johansen R.F., Seeberg E. (2002). AlkB-Mediated Oxidative Demethylation Reverses DNA Damage in *Escherichia coli*. Nature.

[10] Trewick S.C., Henshaw T.F., Hausinger R.P., Lindahl T., Sedgwick B. (2002). Oxidative Demethylation by *Escherichia coli* AlkB Directly Reverts DNA Base Damage. Nature.

[11] Ougland R., Rognes T., Klungland A., Larsen E. (2015). Non-Homologous Functions of the AlkB Homologs. J. Mol. Cell Biol..

[12] Yi C., He C. (2013). DNA Repair by Reversal of DNA Damage. Cold Spring Harb. Perspect. Biol..

[13] Duncan T., Trewick S.C., Koivisto P., Bates P.A., Lindahl T., Sedgwick B. (2002). Reversal of DNA Alkylation Damage by Two Human Dioxygenases. Proc. Natl. Acad. Sci. USA.

[14] Gerken T., Girard C.A., Tung Y.-C.L., Webby C.J., Saudek V., Hewitson K.S., Yeo G.S.H., McDonough M.A., Cunliffe S., McNeill L.A. (2007). The Obesity-Associated FTO Gene Encodes a 2-Oxoglutarate-Dependent Nucleic Acid Demethylase. Science.

[15] Kurowski M.A., Bhagwat A.S., Papaj G., Bujnicki J.M. (2003). Phylogenomic Identification of Five New Human Homologs of the DNA Repair Enzyme AlkB. BMC Genom..

[16] Wei Y.F., Carter K.C., Wang R.P., Shell B.K. (1996). Molecular Cloning and Functional Analysis of a Human cDNA Encoding an *Escherichia coli* AlkB Homolog, a Protein Involved in DNA Alkylation Damage Repair. Nucleic Acids Res..

[17] Li Q., Zhu Q. (2023). The Role of Demethylase AlkB Homologs in Cancer. Front. Oncol..

[18] He P.C., He C. (2021). M6 A RNA Methylation: From Mechanisms to Therapeutic Potential. EMBO J..

[19] Qu J., Yan H., Hou Y., Cao W., Liu Y., Zhang E., He J., Cai Z. (2022). RNA Demethylase ALKBH5 in Cancer: From Mechanisms to Therapeutic Potential. J. Hematol. Oncol..

[20] Wang J., Wang J., Gu Q., Ma Y., Yang Y., Zhu J., Zhang Q. (2020). The Biological Function of m6A Demethylase ALKBH5 and Its Role in Human Disease. Cancer Cell Int..

[21] Bian K., Lenz S.A.P., Tang Q., Chen F., Qi R., Jost M., Drennan C.L., Essigmann J.M., Wetmore S.D., Li D. (2019). DNA Repair Enzymes ALKBH2, ALKBH3, and AlkB Oxidize 5-Methylcytosine to 5-Hydroxymethylcytosine, 5-Formylcytosine and 5-Carboxylcytosine In Vitro. Nucleic Acids Res..

[22] Falnes P. (2004). Repair of 3-Methylthymine and 1-Methylguanine Lesions by Bacterial and Human AlkB Proteins. Nucleic Acids Res..

[23] Ma J., Qi R., Harcourt E.M., Chen Y.-T., Barbosa G.M., Peng Z., Howarth S., Delaney S., Li D. (2024). 3,N4-Etheno-5-Methylcytosine Blocks TET1-3 Oxidation but Is Repaired by ALKBH2, 3 and FTO. Nucleic Acids Res..

[24] Ringvoll J., Moen M.N., Nordstrand L.M., Meira L.B., Pang B., Bekkelund A., Dedon P.C., Bjelland S., Samson L.D., Falnes P.Ø. (2008). AlkB Homologue 2–Mediated Repair of Ethenoadenine Lesions in Mammalian DNA. Cancer Res..

[25] Ringvoll J., Nordstrand L.M., Vagbo C.B., Talstad V., Reite K., Aas P.A., Lauritzen K.H., Liabakk N.B., Bjork A., Doughty R.W. (2006). Repair Deficient Mice Reveal mABH2 as the Primary Oxidative Demethylase for Repairing 1meA and 3meC Lesions in DNA. EMBO J..

[26] You C., Wang P., Nay S.L., Wang J., Dai X., O’Connor T.R., Wang Y. (2016). Roles of Aag, Alkbh2, and Alkbh3 in the Repair of Carboxymethylated and Ethylated Thymidine Lesions. ACS Chem. Biol..

[27] Lee D.H., Jin S.G., Cai S., Chen Y., Pfeifer G.P., O’Connor T.R. (2005). Repair of Methylation Damage in DNA and RNA by Mammalian AlkB Homologues. J. Biol. Chem..

[28] Yang C.G., Yi C., Duguid E.M., Sullivan C.T., Jian X., Rice P.A., He C. (2008). Crystal Structures of DNA/RNA Repair Enzymes AlkB and ABH2 Bound to dsDNA. Nature.

[29] Yi C., Chen B., Qi B., Zhang W., Jia G., Zhang L., Li C.J., Dinner A.R., Yang C.-G., He C. (2012). Duplex Interrogation by a Direct DNA Repair Protein in Search of Base Damage. Nat. Struct. Mol. Biol..

[30] Zheng G., Fu Y., He C. (2014). Nucleic Acid Oxidation in DNA Damage Repair and Epigenetics. Chem. Rev..

[31] Clifton I.J., McDonough M.A., Ehrismann D., Kershaw N.J., Granatino N., Schofield C.J. (2006). Structural Studies on 2-Oxoglutarate Oxygenases and Related Double-Stranded Beta-Helix Fold Proteins. J. Inorg. Biochem..

[32] Waheed S.O., Ramanan R., Chaturvedi S.S., Lehnert N., Schofield C.J., Christov C.Z., Karabencheva-Christova T.G. (2020). Role of Structural Dynamics in Selectivity and Mechanism of Non-Heme Fe(II) and 2-Oxoglutarate-Dependent Oxygenases Involved in DNA Repair. ACS Cent. Sci..

[33] Johannessen T.-C.A., Prestegarden L., Grudic A., Hegi M.E., Tysnes B.B., Bjerkvig R. (2013). The DNA Repair Protein ALKBH2 Mediates Temozolomide Resistance in Human Glioblastoma Cells. Neuro Oncol..

[34] Kaina B., Christmann M. (2019). DNA Repair in Personalized Brain Cancer Therapy with Temozolomide and Nitrosoureas. DNA Repair.

[35] Shimada K., Nakamura M., Anai S., De Velasco M., Tanaka M., Tsujikawa K., Ouji Y., Konishi N. (2009). A Novel Human AlkB Homologue, ALKBH8, Contributes to Human Bladder Cancer Progression. Cancer Res..

[36] Ueda Y., Ooshio I., Fusamae Y., Kitae K., Kawaguchi M., Jingushi K., Hase H., Harada K., Hirata K., Tsujikawa K. (2017). AlkB Homolog 3-Mediated tRNA Demethylation Promotes Protein Synthesis in Cancer Cells. Sci. Rep..

[37] Claussnitzer M., Dankel S.N., Kim K.-H., Quon G., Meuleman W., Haugen C., Glunk V., Sousa I.S., Beaudry J.L., Puviindran V. (2015). FTO Obesity Variant Circuitry and Adipocyte Browning in Humans. N. Engl. J. Med..

[38] Dina C., Meyre D., Gallina S., Durand E., Korner A., Jacobson P., Carlsson L.M.S., Kiess W., Vatin V., Lecoeur C. (2007). Variation in FTO Contributes to Childhood Obesity and Severe Adult Obesity. Nat. Genet..

[39] Frayling T.M., Timpson N.J., Weedon M.N., Zeggini E., Freathy R.M., Lindgren C.M., Perry J.R.B., Elliott K.S., Lango H., Rayner N.W. (2007). A Common Variant in the FTO Gene Is Associated with Body Mass Index and Predisposes to Childhood and Adult Obesity. Science.

[40] Smemo S., Tena J.J., Kim K.-H., Gamazon E.R., Sakabe N.J., Gómez-Marín C., Aneas I., Credidio F.L., Sobreira D.R., Wasserman N.F. (2014). Obesity-Associated Variants within FTO form Long-Range Functional Connections with IRX3. Nature.

[41] Freathy R.M., Timpson N.J., Lawlor D.A., Pouta A., Ben-Shlomo Y., Ruokonen A., Ebrahim S., Shields B., Zeggini E., Weedon M.N. (2008). Common Variation in the FTO Gene Alters Diabetes-Related Metabolic Traits to the Extent Expected given Its Effect on BMI. Diabetes.

[42] Scuteri A., Sanna S., Chen W.-M., Uda M., Albai G., Strait J., Najjar S., Nagaraja R., Orrú M., Usala G. (2007). Genome-Wide Association Scan Shows Genetic Variants in the FTO Gene Are Associated with Obesity-Related Traits. PLoS Genet..

[43] Keller L., Xu W., Wang H.-X., Winblad B., Fratiglioni L., Graff C. (2011). The Obesity Related Gene, FTO, Interacts with APOE, and Is Associated with Alzheimer’s Disease Risk: A Prospective Cohort Study. J. Alzheimers Dis..

[44] Reitz C., Tosto G., Mayeux R., Luchsinger J.A., NIA-LOAD/NCRAD Family Study Group, Alzheimer’s Disease Neuroimaging Initiative (2012). Genetic Variants in the Fat and Obesity Associated (FTO) Gene and Risk of Alzheimer’s Disease. PLoS ONE.

[45] Lim A., Zhou J., Sinha R.A., Singh B.K., Ghosh S., Lim K.-H., Chow P.K.-H., Woon E.C.Y., Yen P.M. (2016). Hepatic FTO Expression Is Increased in NASH and Its Silencing Attenuates Palmitic Acid-Induced Lipotoxicity. Biochem. Biophys Res. Commun..

[46] Li Y., Zheng D., Wang F., Xu Y., Yu H., Zhang H. (2019). Expression of Demethylase Genes, FTO and ALKBH1, Is Associated with Prognosis of Gastric Cancer. Dig. Dis. Sci..

[47] Xie Q., Wu T.P., Gimple R.C., Li Z., Prager B.C., Wu Q., Yu Y., Wang P., Wang Y., Gorkin D.U. (2018). N6-Methyladenine DNA Modification in Glioblastoma. Cell.

[48] Calvo J.A., Meira L.B., Lee C.-Y.I., Moroski-Erkul C.A., Abolhassani N., Taghizadeh K., Eichinger L.W., Muthupalani S., Nordstrand L.M., Klungland A. (2012). DNA Repair Is Indispensable for Survival after Acute Inflammation. J. Clin. Investig..

[49] Aas P.A., Otterlei M., Falnes P., Vågbø C.B., Skorpen F., Akbari M., Sundheim O., Bjørås M., Slupphaug G., Seeberg E. (2003). Human and Bacterial Oxidative Demethylases Repair Alkylation Damage in Both RNA and DNA. Nature.

[50] Xu B., Liu D., Wang Z., Tian R., Zuo Y. (2021). Multi-Substrate Selectivity Based on Key Loops and Non-Homologous Domains: New Insight into ALKBH Family. Cell. Mol. Life Sci..

[51] Wu S., Xu W., Liu S., Chen B., Wang X., Wang Y., Liu S., Wu J. (2011). Down-Regulation of ALKBH2 Increases Cisplatin Sensitivity in H1299 Lung Cancer Cells. Acta Pharmacol. Sin..

[52] Cetica V., Genitori L., Giunti L., Sanzo M., Bernini G., Massimino M., Sardi I. (2009). Pediatric Brain Tumors: Mutations of Two Dioxygenases (hABH2 and hABH3) That Directly Repair Alkylation Damage. J. Neurooncol..

[53] Fujii T., Shimada K., Anai S., Fujimoto K., Konishi N. (2013). ALKBH2, a Novel AlkB Homologue, Contributes to Human Bladder Cancer Progression by Regulating MUC1 Expression. Cancer Sci..

[54] Gao W., Li L., Xu P., Fang J., Xiao S., Chen S. (2011). Frequent Down-Regulation of hABH2 in Gastric Cancer and Its Involvement in Growth of Cancer Cells. J. Gastroenterol. Hepatol..

[55] Ke B., Ye K., Cheng S. (2020). ALKBH2 Inhibition Alleviates Malignancy in Colorectal Cancer by Regulating BMI1-Mediated Activation of NF-κB Pathway. World J. Surg. Oncol..

[56] Collins F.S., Brooks L.D., Chakravarti A. (1998). A DNA Polymorphism Discovery Resource for Research on Human Genetic Variation. Genome Res..

[57] Fu D., Samson L.D., Hübscher U., van Loon B. (2015). The Interaction between ALKBH2 DNA Repair Enzyme and PCNA Is Direct, Mediated by the Hydrophobic Pocket of PCNA and Perturbed in Naturally-Occurring ALKBH2 Variants. DNA Repair.

[58] Cho H., Shiraishi K., Sunami K., Momozawa Y., Yoshida T., Matsumoto S., Matsuda K., Saito M., Goto A., Honda T. (2025). Genomic Profiles of Pathogenic and Moderate-Penetrance Germline Variants Associated with Risk of Early-Onset Lung Adenocarcinoma. J. Thorac. Oncol..

[59] Ng P.C., Henikoff S. (2002). Accounting for Human Polymorphisms Predicted to Affect Protein Function. Genome Res..

[60] Ng P.C., Henikoff S. (2001). Predicting Deleterious Amino Acid Substitutions. Genome Res..

[61] Adzhubei I.A., Schmidt S., Peshkin L., Ramensky V.E., Gerasimova A., Bork P., Kondrashov A.S., Sunyaev S.R. (2010). A Method and Server for Predicting Damaging Missense Mutations. Nat. Methods.

[62] Kircher M., Witten D.M., Jain P., O’Roak B.J., Cooper G.M., Shendure J. (2014). A General Framework for Estimating the Relative Pathogenicity of Human Genetic Variants. Nat. Genet..

[63] Ioannidis N.M., Rothstein J.H., Pejaver V., Middha S., McDonnell S.K., Baheti S., Musolf A., Li Q., Holzinger E., Karyadi D. (2016). REVEL: An Ensemble Method for Predicting the Pathogenicity of Rare Missense Variants. Am. J. Hum. Genet..

[64] Dong C., Wei P., Jian X., Gibbs R., Boerwinkle E., Wang K., Liu X. (2015). Comparison and Integration of Deleteriousness Prediction Methods for Nonsynonymous SNVs in Whole Exome Sequencing Studies. Hum. Mol. Genet..

[65] Cheng J., Novati G., Pan J., Bycroft C., Žemgulytė A., Applebaum T., Pritzel A., Wong L.H., Zielinski M., Sargeant T. (2023). Accurate Proteome-Wide Missense Variant Effect Prediction with AlphaMissense. Science.

[66] Choi Y., Chan A.P. (2015). PROVEAN Web Server: A Tool to Predict the Functional Effect of Amino Acid Substitutions and Indels. Bioinformatics.

[67] Capriotti E., Calabrese R., Fariselli P., Martelli P.L., Altman R.B., Casadio R. (2013). WS-SNPs&GO: A Web Server for Predicting the Deleterious Effect of Human Protein Variants Using Functional Annotation. BMC Genom..

[68] Tang H., Thomas P.D. (2016). PANTHER-PSEP: Predicting Disease-Causing Genetic Variants Using Position-Specific Evolutionary Preservation. Bioinformatics.

[69] Bendl J., Stourac J., Salanda O., Pavelka A., Wieben E.D., Zendulka J., Brezovsky J., Damborsky J. (2014). PredictSNP: Robust and Accurate Consensus Classifier for Prediction of Disease-Related Mutations. PLoS Comput. Biol..

[70] Capriotti E., Calabrese R., Casadio R. (2006). Predicting the Insurgence of Human Genetic Diseases Associated to Single Point Protein Mutations with Support Vector Machines and Evolutionary Information. Bioinformatics.

[71] Garcia F.A.d.O., Andrade E.S.d., Palmero E.I. (2022). Insights on Variant Analysis in Silico Tools for Pathogenicity Prediction. Front. Genet..

[72] Mottaz A., David F.P.A., Veuthey A.-L., Yip Y.L. (2010). Easy Retrieval of Single Amino-Acid Polymorphisms and Phenotype Information Using SwissVar. Bioinformatics.

[73] Suybeng V., Koeppel F., Harlé A., Rouleau E. (2020). Comparison of Pathogenicity Prediction Tools on Somatic Variants. J. Mol. Diagn..

[74] Liu X., Wu C., Li C., Boerwinkle E. (2016). dbNSFP v3.0: A One-Stop Database of Functional Predictions and Annotations for Human Non-Synonymous and Splice Site SNVs. Hum. Mutat..

[75] Bendl J., Musil M., Štourač J., Zendulka J., Damborský J., Brezovský J. (2016). PredictSNP2: A Unified Platform for Accurately Evaluating SNP Effects by Exploiting the Different Characteristics of Variants in Distinct Genomic Regions. PLoS Comput. Biol..

[76] Ng P.C., Henikoff S. (2003). SIFT: Predicting Amino Acid Changes That Affect Protein Function. Nucleic Acids Res..

[77] Wang D., Li J., Wang Y., Wang E. (2022). A Comparison on Predicting Functional Impact of Genomic Variants. NAR Genom. Bioinform..

[78] Choudhury A., Mohammad T., Anjum F., Shafie A., Singh I.K., Abdullaev B., Pasupuleti V.R., Adnan M., Yadav D.K., Hassan M.I. (2022). Comparative Analysis of Web-Based Programs for Single Amino Acid Substitutions in Proteins. PLoS ONE.

[79] Laskar R., Ali S. (2021). Mutational Analysis and Assessment of Its Impact on Proteins of SARS-CoV-2 Genomes from India. Gene.

[80] Mi H., Huang X., Muruganujan A., Tang H., Mills C., Kang D., Thomas P.D. (2017). PANTHER Version 11: Expanded Annotation Data from Gene Ontology and Reactome Pathways, and Data Analysis Tool Enhancements. Nucleic Acids Res..

[81] Bhatnager R., Dang A.S. (2018). Comprehensive In-Silico Prediction of Damage Associated SNPs in Human Prolidase Gene. Sci. Rep..

[82] Capriotti E., Fariselli P. (2023). PhD-SNPg: Updating a Webserver and Lightweight Tool for Scoring Nucleotide Variants. Nucleic Acids Res..

[83] Dingerdissen H.M., Torcivia-Rodriguez J., Hu Y., Chang T.-C., Mazumder R., Kahsay R. (2018). BioMuta and BioXpress: Mutation and Expression Knowledgebases for Cancer Biomarker Discovery. Nucleic Acids Res..

[84] Pan Y., Karagiannis K., Zhang H., Dingerdissen H., Shamsaddini A., Wan Q., Simonyan V., Mazumder R. (2014). Human Germline and Pan-Cancer Variomes and Their Distinct Functional Profiles. Nucleic Acids Res..

[85] Wu T.-J., Shamsaddini A., Pan Y., Smith K., Crichton D.J., Simonyan V., Mazumder R. (2014). A Framework for Organizing Cancer-Related Variations from Existing Databases, Publications and NGS Data Using a High-Performance Integrated Virtual Environment (HIVE). Database.

[86] Chen B., Liu H., Sun X., Yang C.-G. (2010). Mechanistic Insight into the Recognition of Single-Stranded and Double-Stranded DNA Substrates by ABH2 and ABH3. Mol. Biosyst..

[87] Davletgildeeva A.T., Tyugashev T.E., Zhao M., Ishchenko A.A., Saparbaev M., Kuznetsov N.A. (2025). Role of Individual Amino Acid Residues Directly Involved in Damage Recognition in Active Demethylation by ABH2 Dioxygenase. Int. J. Mol. Sci..

[88] Yu B., Edstrom W.C., Benach J., Hamuro Y., Weber P.C., Gibney B.R., Hunt J.F. (2006). Crystal Structures of Catalytic Complexes of the Oxidative DNA/RNA Repair Enzyme AlkB. Nature.

[89] Davletgildeeva A.T., Tyugashev T.E., Zhao M., Kuznetsov N.A., Ishchenko A.A., Saparbaev M., Kuznetsova A.A. (2023). Individual Contributions of Amido Acid Residues Tyr122, Ile168, and Asp173 to the Activity and Substrate Specificity of Human DNA Dioxygenase ABH2. Cells.

