# Supplementary material for: Predictive Analysis and Validation of Critical Missense SNPs of the ABH2 Gene Using Structural Bioinformatics
Source: Int J Mol Sci. 2025 Nov 29;26(23):11593. doi: 10.3390/ijms262311593 (PMC12692101; doi:10.3390/ijms262311593)
Supplement: Supplementary file 1 [file ijms-26-11593-s001.zip › ijms-3992794-supplementary.pdf]

Supplementary materials

Predictive Analysis and Validation of Critical Missense SNPs of the *ABH2* Gene Using Structural Bioinformatics

Anastasiia T. Davletgildeeva [1,*], Timofey E. Tyugashev [1], Viktoriia V. Sagalakova [1], Mingxing Zhao [2] and Nikita A. Kuznetsov [1,2,*]

[1] *Institute of Chemical Biology and Fundamental Medicine, Siberian Branch of Russian Academy of Sciences, 630090 Novosibirsk, Russia*

[2] *Department of Natural Sciences, Novosibirsk State University, 630090 Novosibirsk, Russia*

This document describes the algorithms used to predict the functional effects of single nucleotide polymorphisms (SNPs) on the ABH2 enzyme.

SIFT

SIFT (Sorting Intolerant From Tolerant) is a widely used bioinformatic tool designed to predict the impact of nonsynonymous SNPs—those causing amino acid substitutions—on protein function [1]. It focuses on nonsynonymous SNPs that alter the amino acid sequence of the encoded protein and are potentially associated with diseases or phenotype changes. When analyzing the potential deleteriousness of a particular amino acid substitution, SIFT uses data on its conservation among close homologs. If a position is highly conserved and shows little variation between species, SIFT predicts that substitutions there may disrupt protein function. The identifier numbers (rs) of 231 identified ABH2 SNPs were analyzed using SIFT, and the program identified 142 of them as potentially deleterious to the protein ("deleterious").

PolyPhen2

PolyPhen2 predicts the potential effects of SNP-induced amino acid substitutions on protein structure and function by considering multiple criteria, including the conservation of the specific amino acid residue within the protein sequence, as well as the existing information on protein structure and sequence. Additionally, the physicochemical properties of both the original and substituted residues are taken into account [2]. Of the 231 SNPs analyzed in this study, 78 substitutions were classified as "probably damaging," 30 as "possibly damaging," and the remaining 123 as "benign."

CADD

CADD (Combined Annotation Dependent Depletion) is a comprehensive tool for predicting the functional impact of various SNPs, including nonsynonymous variants. CADD integrates numerous genomic annotations—such as sequence conservation, regulatory elements, structural data, and epigenetic marks—into a single machine learning model. Trained on observed (presumed neutral) variants and synthetically generated (presumed deleterious) variants, CADD provides a quantitative score reflecting the likelihood that a variant is deleterious. CADD is one of the most widely used and cited tools for variant pathogenicity assessment and demonstrates high sensitivity and specificity owing to its integration of extensive genomic and functional data [3]. Among the 231 SNPs examined in this work, CADD classified 16 as "likely deleterious."

REVEL

REVEL is an ensemble machine learning method for predicting the pathogenicity of missense variants in the human genome. It combines predictions from multiple individual algorithms—including PolyPhen2, SIFT, PROVEAN, and others—while also incorporating conservation metrics and functional annotations [4]. REVEL is further trained on rare pathogenic

variants not represented in the databases used by the constituent tools [5]. One advantage of REVEL is that its predictions outperform those of most individual and other ensemble methods, particularly for rare variants with allele frequencies below 0.5% [6]. In our analysis of 231 ABH2 SNPs, REVEL designated 31 variants as “likely disease-causing.”

MetaLR

MetaLR, like REVEL, is an ensemble approach that integrates nine independent deleteriousness scores, including SIFT, PolyPhen2, GERP++, MutationTaster, Mutation Assessor, FATHMM, LRT, SiPhy, and PhyloP, together with allele frequency data via logistic regression to improve variant classification accuracy. The resulting MetaLR score ranges from 0 to 1, with higher values indicating increased likelihood of a damaging effect. MetaLR predictions have been shown to surpass the accuracy of many individual tools such as SIFT and PolyPhen2 [7]. In this study, MetaLR flagged 5 of the 231 ABH2 SNPs as potentially deleterious.

AlphaMissense

AlphaMissense is a modern artificial intelligence tool developed by DeepMind for predicting the pathogenicity of all possible missense variants in human proteins. It employs deep learning techniques, akin to those used in AlphaFold, to analyze protein sequences and structures and estimates variant pathogenicity based on log-likelihood differences. A key strength of AlphaMissense lies in its incorporation of three-dimensional structural context for each residue, enhancing prediction accuracy. However, predictive reliability varies by gene [8]. In our analysis of 231 nonsynonymous ABH2 variants, AlphaMissense identified 61 as “likely pathogenic” and 17 as “ambiguous.”

PROVEAN

PROVEAN uses delta analysis of homologous and distant sequences from the NCBI NR database using BLASTP to prepare its predictions of the potential harmfulness of amino acid substitutions [9]. Sequence redundancy is reduced through clustering, and a delta score is calculated based on changes in sequence similarity using the BLOSUM62 substitution matrix [10]. PROVEAN’s accuracy is comparable to that of SIFT and PolyPhen-2, but optimal predictions are often obtained by combining PROVEAN with tools that incorporate structural data, as PROVEAN may be less reliable for proteins with rare or unique sequences [11,12]. PROVEAN classified 108 of the 231 ABH2 nonsynonymous SNPs as “damaging.”

PANTHER

PANTHER predicts the functional impact of SNP-induced amino acid substitutions based on evolutionary and functional protein data. The key algorithm that PANTHER uses to assess the impact of nonsynonymous amino acid substitutions caused by SNPs is the Position-Specific Evolutionary Preservation (PSEP) method. PANTHER reconstructs a phylogenetic tree for protein

families and constructs probabilistic models of ancestral sequences for each node in the tree. PSEP assesses the duration a residue has been preserved across ancestors to infer variant impact, combining evolutionary, structural, and functional information, including hidden Markov models of protein domains [13]. At the same time, among the disadvantages of using PANTHER to predict the consequences of amino acid substitutions on protein functions, one can highlight the limited coverage of analyzed variants and the increased level of false negative results and underestimation of the harmfulness of some substitutions compared to many other programs. [14,15]. In this study, PANTHER analyzed 204 of the 231 ABH2 SNPs, categorizing 98 as “probably damaging” and 34 as “possibly damaging.”

WS-SNPs&GO

The operating principle of the WS-SNPs&GO program for prediction of the pathological effect of SNP-induced substitutions of amino acid residues is based on machine learning methods, in particular Support Vector Machines (SVM), and the use of information on the functional annotation of proteins, represented through gene ontology terms (Gene Ontology, GO) [16]. One of the key advantages of this program is that it uses data on the immediate environment of the replaced amino acid residue and its surface accessibility to create a prediction, if the three-dimensional structure of the protein being analyzed is available. Moreover, the use of functional annotation of a protein in the form of GO terms allows WS-SNPs&GO to take into account characteristics such as molecular function, role in the organism, and localization of the protein in the cell. On the other hand, the requirement of the data on specific changes in the protein and its 3D structure may limit the analysis in the absence of detailed information.

Because part of the ABH2 protein's peptide sequence is unstructured, the amino acid residues within this region were analyzed using WS-SNPs&GO without regard to structural data. As a result, of the 231 ABH2 SNPs analyzed that resulted in nonsynonymous substitutions, 36 were classified by WS-SNPs&GO as potentially pathological (“disease”).

PhD SNP

PhD-SNP is another program for predicting the negative effects of SNPs on protein functionality, the operating principle of which is based on the SVM machine learning method. [17]. PhD-SNP uses information about the protein sequence and the degree of conservation of the positions of specific amino acid residues based on multiple sequence alignment when preparing a prediction. The program was trained on a large dataset of known pathogenic and neutral variants using SVM for their classification. The results obtained using PhD-SNP are considered well-balanced in terms of the ratio of false positives to false negatives. However, in the absence of structural data, the reliability of PhD-SNP predictions may be reduced. After analyzing 231 nonsynonymous ABH2 SNP variants, this program classified 85 variants as “deleterious.”

PredictSNP

PredictSNP combines the results of several programs to predict the consequences of amino acid substitutions in a protein and forms its own assessment of potential harmfulness taking into account the confidence estimates of each of the integrated methods [18]. Among others PredictSNP takes into account data from programs such as SIFT, PhD-SNP, and PolyPhen2. The method is trained using various machine learning algorithms, including Naive Bayes, regression, neural networks, SVM, k-nearest neighbors, and random forest. By using a consensus approach, the prediction results obtained using PredictSNP show higher accuracy than those obtained using individual tools [19]. In an analysis of 231 ABH2 SNP variants, 86 were classified as “deleterious” according to PredictSNP.

References

1. Ng, P.C.; Henikoff, S. SIFT: Predicting Amino Acid Changes That Affect Protein Function. *Nucleic Acids Res* **2003**, *31*, 3812–3814, doi:10.1093/nar/gkg509.
2. Bhatnager, R.; Dang, A.S. Comprehensive In-Silico Prediction of Damage Associated SNPs in Human Prolidase Gene. *Scientific Reports* **2018**, *8*, 9430, doi:10.1038/s41598-018-27789-0.
3. Kircher, M.; Witten, D.M.; Jain, P.; O’Roak, B.J.; Cooper, G.M.; Shendure, J. A General Framework for Estimating the Relative Pathogenicity of Human Genetic Variants. *Nat Genet* **2014**, *46*, 310–315, doi:10.1038/ng.2892.
4. Garcia, F.A. de O.; Andrade, E.S. de; Palmero, E.I. Insights on Variant Analysis in Silico Tools for Pathogenicity Prediction. *Frontiers in Genetics* **2022**, *Volume 13-2022*.
5. Mottaz, A.; David, F.P.A.; Veuthey, A.-L.; Yip, Y.L. Easy Retrieval of Single Amino-Acid Polymorphisms and Phenotype Information Using SwissVar. *Bioinformatics* **2010**, *26*, 851–852, doi:10.1093/bioinformatics/btq028.
6. Suybeng, V.; Koeppel, F.; Harlé, A.; Rouleau, E. Comparison of Pathogenicity Prediction Tools on Somatic Variants. *The Journal of Molecular Diagnostics* **2020**, *22*, 1383–1392, doi:10.1016/j.jmoldx.2020.08.007.
7. Liu, X.; Wu, C.; Li, C.; Boerwinkle, E. dbNSFP v3.0: A One-Stop Database of Functional Predictions and Annotations for Human Non-Synonymous and Splice Site SNVs. *Hum Mutat* **2016**, *37*, 235–241, doi:10.1002/humu.22932.
8. Cheng, J.; Novati, G.; Pan, J.; Bycroft, C.; Žemgulytė, A.; Applebaum, T.; Pritzel, A.; Wong, L.H.; Zielinski, M.; Sargeant, T.; et al. Accurate Proteome-Wide Missense Variant Effect Prediction with AlphaMissense. *Science* **2023**, *381*, eadg7492, doi:10.1126/science.adg7492.
9. Choi, Y.; Chan, A.P. PROVEAN Web Server: A Tool to Predict the Functional Effect of Amino Acid Substitutions and Indels. *Bioinformatics* **2015**, *31*, 2745–2747, doi:10.1093/bioinformatics/btv195.
10. Wang, D.; Li, J.; Wang, Y.; Wang, E. A Comparison on Predicting Functional Impact of Genomic Variants. *NAR Genom Bioinform* **2022**, *4*, lqab122, doi:10.1093/nargab/lqab122.
11. Choudhury, A.; Mohammad, T.; Anjum, F.; Shafie, A.; Singh, I.K.; Abdullaev, B.; Pasupuleti, V.R.; Adnan, M.; Yadav, D.K.; Hassan, Md.I. Comparative Analysis of Web-Based Programs for Single Amino Acid Substitutions in Proteins. *PLoS One* **2022**, *17*, e0267084, doi:10.1371/journal.pone.0267084.
12. Laskar, R.; Ali, S. Mutational Analysis and Assessment of Its Impact on Proteins of SARS-CoV-2 Genomes from India. *Gene* **2021**, *778*, 145470, doi:10.1016/j.gene.2021.145470.

13. Mi, H.; Huang, X.; Muruganujan, A.; Tang, H.; Mills, C.; Kang, D.; Thomas, P.D. PANTHER Version 11: Expanded Annotation Data from Gene Ontology and Reactome Pathways, and Data Analysis Tool Enhancements. *Nucleic Acids Research* **2017**, *45*, D183–D189, doi:10.1093/nar/gkw1138.
14. Dong, C.; Wei, P.; Jian; Gibbs, R.; Boerwinkle, E.; Wang, K.; Liu, X. Comparison and Integration of Deleteriousness Prediction Methods for Nonsynonymous SNVs in Whole Exome Sequencing Studies | Human Molecular Genetics | Oxford Academic. *Human Molecular Genetics* **2015**, *24*, 2125–2137, doi:10.1093/hmg/ddu733.
15. Tang, H.; Thomas, P.D. PANTHER-PSEP: Predicting Disease-Causing Genetic Variants Using Position-Specific Evolutionary Preservation. *Bioinformatics* **2016**, *32*, 2230–2232, doi:10.1093/bioinformatics/btw222.
16. Capriotti, E.; Calabrese, R.; Fariselli, P.; Martelli, P.L.; Altman, R.B.; Casadio, R. WS-SNPs&GO: A Web Server for Predicting the Deleterious Effect of Human Protein Variants Using Functional Annotation. *BMC Genomics* **2013**, *14 Suppl 3*, S6, doi:10.1186/1471-2164-14-S3-S6.
17. Capriotti, E.; Fariselli, P. PhD-SNPg: Updating a Webserver and Lightweight Tool for Scoring Nucleotide Variants. *Nucleic Acids Res* **2023**, *51*, W451–W458, doi:10.1093/nar/gkad455.
18. Bendl, J.; Stourac, J.; Salanda, O.; Pavelka, A.; Wieben, E.D.; Zendulka, J.; Brezovsky, J.; Damborsky, J. PredictSNP: Robust and Accurate Consensus Classifier for Prediction of Disease-Related Mutations. *PLoS Comput Biol* **2014**, *10*, e1003440, doi:10.1371/journal.pcbi.1003440.
19. Bendl, J.; Musil, M.; Štourač, J.; Zendulka, J.; Damborský, J.; Brezovský, J. PredictSNP2: A Unified Platform for Accurately Evaluating SNP Effects by Exploiting the Different Characteristics of Variants in Distinct Genomic Regions. *PLoS Comput Biol* **2016**, *12*, e1004962, doi:10.1371/journal.pcbi.1004962.
